# A probabilistic approach for calibration time reduction in hybrid EEG–fTCD brain–computer interfaces

**DOI:** 10.1186/s12938-020-00765-4

**Published:** 2020-04-16

**Authors:** Aya Khalaf, Murat Akcakaya

**Affiliations:** Electrical and Computer Engineering Department, University of Pittsburgh, Pittsburgh, PA USA

**Keywords:** Hybrid brain–computer interfaces, Transfer learning, Common spatial pattern, Template matching, Wavelet decomposition, Probabilistic fusion

## Abstract

**Background:**

Generally, brain–computer interfaces (BCIs) require calibration before usage to ensure efficient performance. Therefore, each BCI user has to attend a certain number of calibration sessions to be able to use the system. However, such calibration requirements may be difficult to fulfill especially for patients with disabilities. In this paper, we introduce a probabilistic transfer learning approach to reduce the calibration requirements of our EEG–fTCD hybrid BCI designed using motor imagery (MI) and flickering mental rotation (MR)/word generation (WG) paradigms. The proposed approach identifies the top similar datasets from previous BCI users to a small training dataset collected from a current BCI user and uses these datasets to augment the training data of the current BCI user. To achieve such an aim, EEG and fTCD feature vectors of each trial were projected into scalar scores using support vector machines. EEG and fTCD class conditional distributions were learnt separately using the scores of each class. Bhattacharyya distance was used to identify similarities between class conditional distributions obtained using training trials of the current BCI user and those obtained using trials of previous users.

**Results:**

Experimental results showed that the performance obtained using the proposed transfer learning approach outperforms the performance obtained without transfer learning for both MI and flickering MR/WG paradigms. In particular, it was found that the calibration requirements can be reduced by at least 60.43% for the MI paradigm, while at most a reduction of 17.31% can be achieved for the MR/WG paradigm.

**Conclusions:**

Data collected using the MI paradigm show better generalization across subjects.

## Background

Noninvasive BCIs are designed mainly to help individuals with limited speech and physical abilities (LSPA) due to neurological deficits to communicate with the surrounding environment without any surgical interventions [1]. Due to its low cost, high temporal resolution, and portability, EEG is the most common neuroimaging modality used to design noninvasive BCIs [1, 2]. However, performance of EEG BCIs degrades due to the low EEG signal-to-noise ratio (SNR) and nonstationarities existing in the EEG signals due to the background brain activity.

Multimodal BCIs have been recently studied to overcome the limitations of EEG-based BCIs and to improve their performance [3]. The most commonly used modality with EEG for hybrid BCI design is functional near-infrared spectroscopy (fNIRS). However, fNIRS lacks the high temporal resolution required for real-time BCI applications [4]. Previously, we have proved that functional transcranial Doppler (fTCD) can be used simultaneously with EEG to design an efficient hybrid EEG–fTCD BCI that outperforms all EEG–fNIRS systems in literature in terms of accuracy and speed [5, 6].

Generally, before being used by each individual, a BCI requires calibration to ensure that it can identify user intent with sufficient accuracy in a reasonable amount of time. Moreover, since the BCI performance is directly proportional to the amount of available training data, each BCI user has to attend a certain number of calibration sessions which may be burdensome for individuals with LSPA.

One potential solution for such a problem is combining data from different BCI users to calibrate the system for a certain user. However, the statistical distribution of the data varies across subjects and even across sessions within the same subject [7]. This limits the transferability of training data across sessions and subjects. The concept of transfer learning focuses on developing algorithms that can improve learning capacity so that the prediction model either learns faster or better on a given dataset through exposure to other datasets [8]. Recently, two categories of transfer learning methods have been studied including domain adaptation and rule adaptation methods [7]. Rule adaptation methods require learning a decision boundary for each subject separately. The decision boundary is considered as a random variable. The distribution of this random variable is found using the decision boundaries estimated based on datasets collected from previous subjects. However, for rule adaptation methods to be efficient, a high number of datasets are needed to estimate the distribution of the decision boundary.

In contrast, domain adaptation approaches have been extensively used for BCIs’ applications. These approaches aim at finding a common structure in the data such that one decision boundary can be generalized across subjects. Finding a common structure can be performed either by finding a linear transformation where the data are invariant across all individuals [9] or using similarity measures to find the most similar datasets to the dataset under test [10]. Data alignment is one of the most common domain adaptation approaches. In a recent study, Zanini et al. [11] proposed aligning the covariance matrices of all sessions and subjects in the Riemannian space to center these covariance matrices with respect to a reference covariance matrix. However, the Riemannian distance (geodesic) computation in the Riemannian space is computationally expensive and unstable. To overcome these limitations, He et al. [12] introduced a method in which, instead of aligning the covariance matrices, they aligned the EEG trials within each session/subject in the Euclidean space based on a reference covariance matrix. However, these alignment methods require high-dimensional covariance matrix estimation which is highly dependent on the number of the available training trials and cannot be performed efficiently with a few number of training trials. In another study, Azab et al. [13] measured the similarity across different feature spaces in which the features were obtained using subject-specific CSP. The measured similarity was used to add a regularization parameter to the objective function of a logistic regression classifier such that the classification parameters are as similar as possible to the parameters of the previous BCI users whose feature spaces are similar to that of the current user. However, this transfer learning approach is not robust for BCIs employing CSP features because extracting features through applying subject-specific CSP yields different feature spaces for different subjects.

Deep learning approaches have been also used to transfer knowledge across different BCI users. For instance, Fahimi et al. [14] used a convolutional neural network (CNN) to learn a general model based on the data from a group of subjects. For a new BCI user, the general CNN model is updated based on a subset of data collected from that new user. In other studies, simultaneous training of an autoencoder and adversarial network was used to learn subject-invariant representations [15, 16]. However, such deep learning approaches require a large number of trials for training.

In this paper, we propose a domain adaptation-based transfer learning approach to reduce the calibration requirements of our hybrid EEG–fTCD BCI utilizing both MI and flickering MR/WG paradigms through transferring BCI training experience. To evaluate the performance of the proposed approach, we formulated 3 binary selection problems for each presentation paradigm including right arm MI versus baseline, left arm MI versus baseline, right versus left arm MI, MR versus baseline, WG versus baseline, and MR versus WG. Common spatial pattern (CSP) and wavelet decomposition were used to extract features from EEG and fTCD data collected using MI paradigm while template matching and wavelet decomposition were used to extract features from EEG and fTCD data of flickering MR/WG paradigm.

To apply transfer learning, similarity between the EEG and fTCD data of the current BCI user and those of the previous users has to be measured. To achieve such aim, we reduced feature vectors of EEG and fTCD data of each trial into scalar SVM scores to learn EEG and fTCD class conditional distributions. Similarities across participants were identified based on these class conditional distributions. In particular, we computed Bhattacharyya distance between the class conditional distributions obtained using the training data of the current BCI user and class conditional distributions obtained using datasets collected from the previous BCI users. After identifying the top similar datasets, we combined the training trials of the current user with trials of these top similar datasets to form a training set that can be used to calibrate the BCI system.

Using the new training set, we evaluated the performance of the system through assessing the test trials of the current BCI user. As mentioned above, for MI paradigm, CSP and wavelet features were extracted while template matching and wavelet features were considered in case of MR/WG paradigm. A probabilistic fusion approach was used to combine EEG and fTCD evidences which were obtained through reducing EEG and fTCD feature vectors of each trial into scalar SVM scores.

## Results

**Fig. 1:**
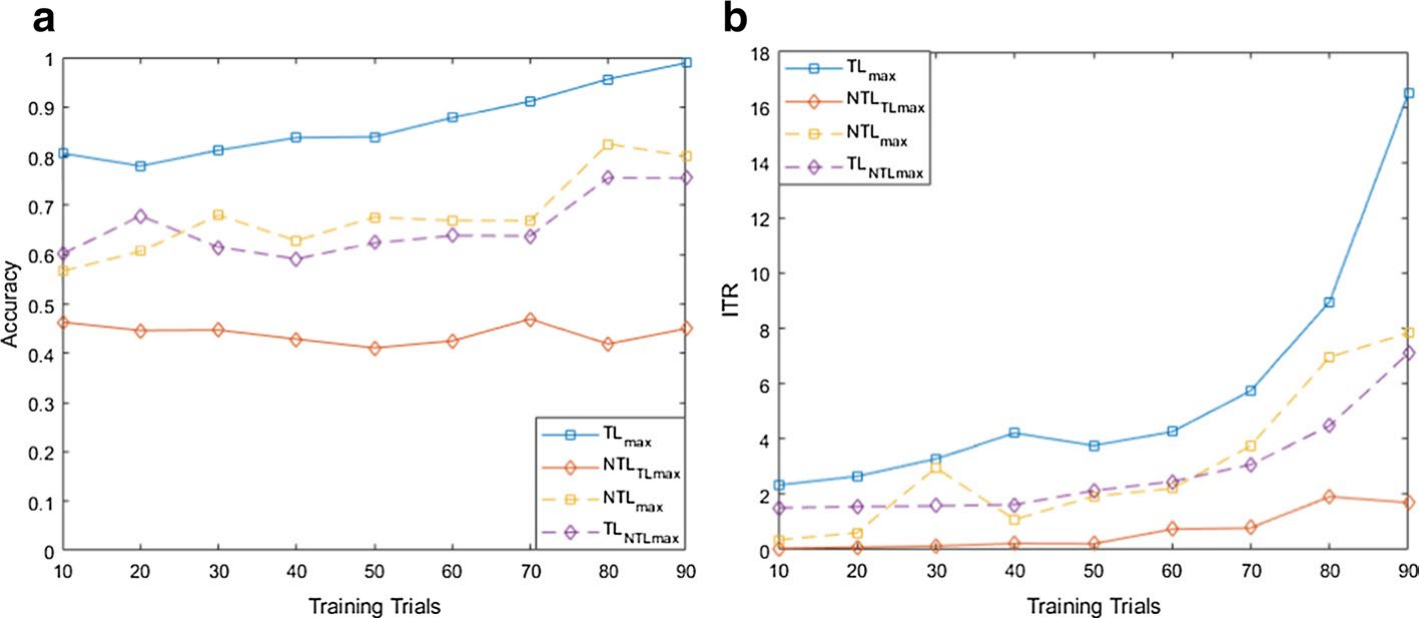
Average accuracy (**a**) and average ITR (**b**) as a function of the number of training trials for right MI versus baseline problem

**Fig. 2:**
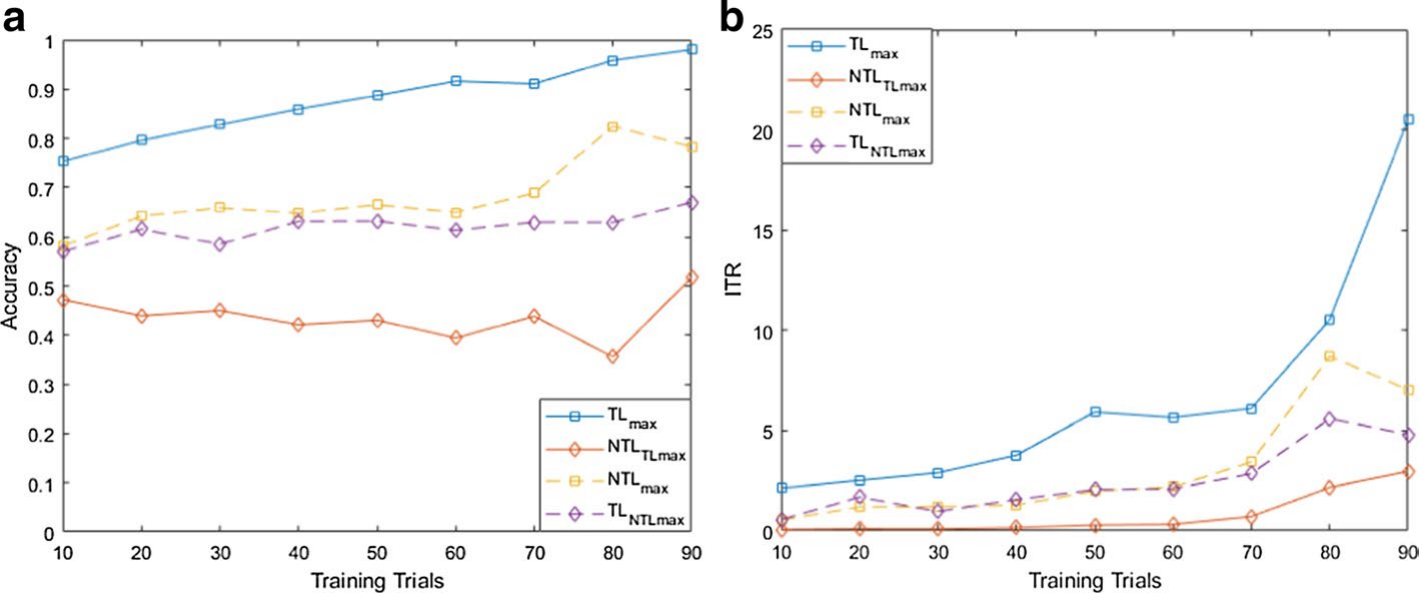
Average accuracy (**a**) and average ITR (**b**) as a function of the number of training trials for left MI versus baseline problem

**Fig. 3:**
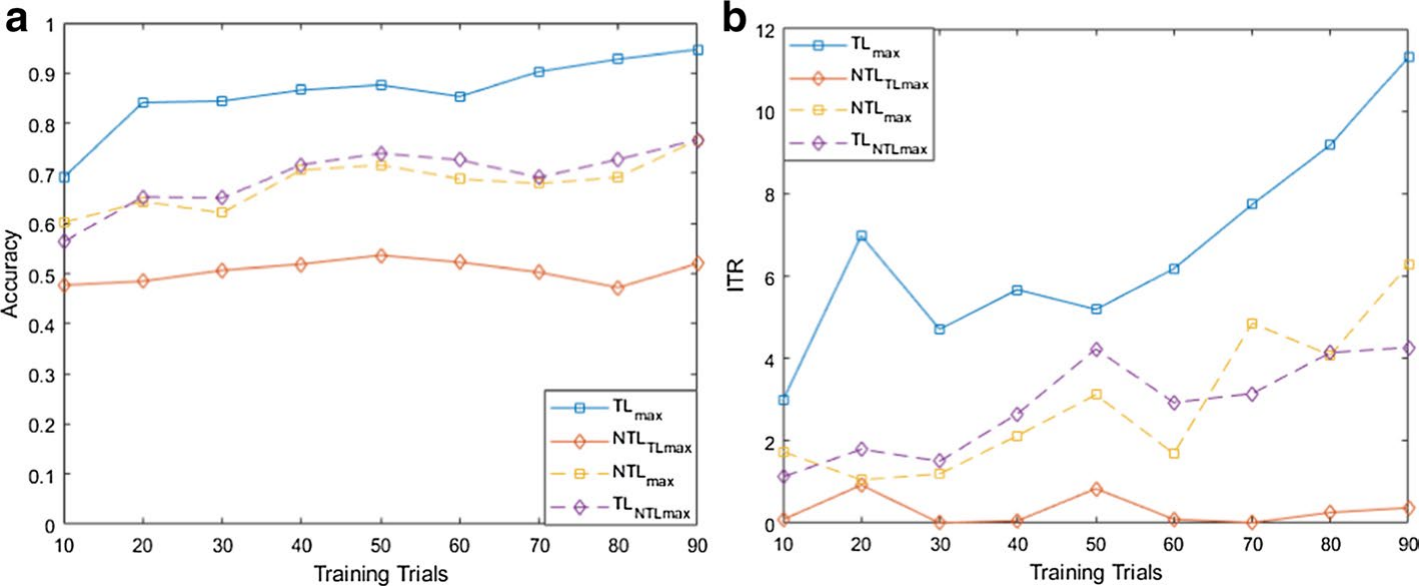
Average accuracy (**a**) and average ITR (**b**) as a function of the number of training trials for right MI versus left MI problem

**Fig. 4:**
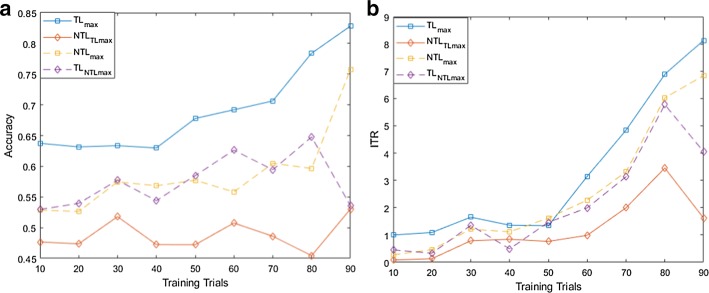
Average accuracy (**a**) and average ITR (**b**) as a function of the number of training trials for MR versus baseline problem

**Fig. 5:**
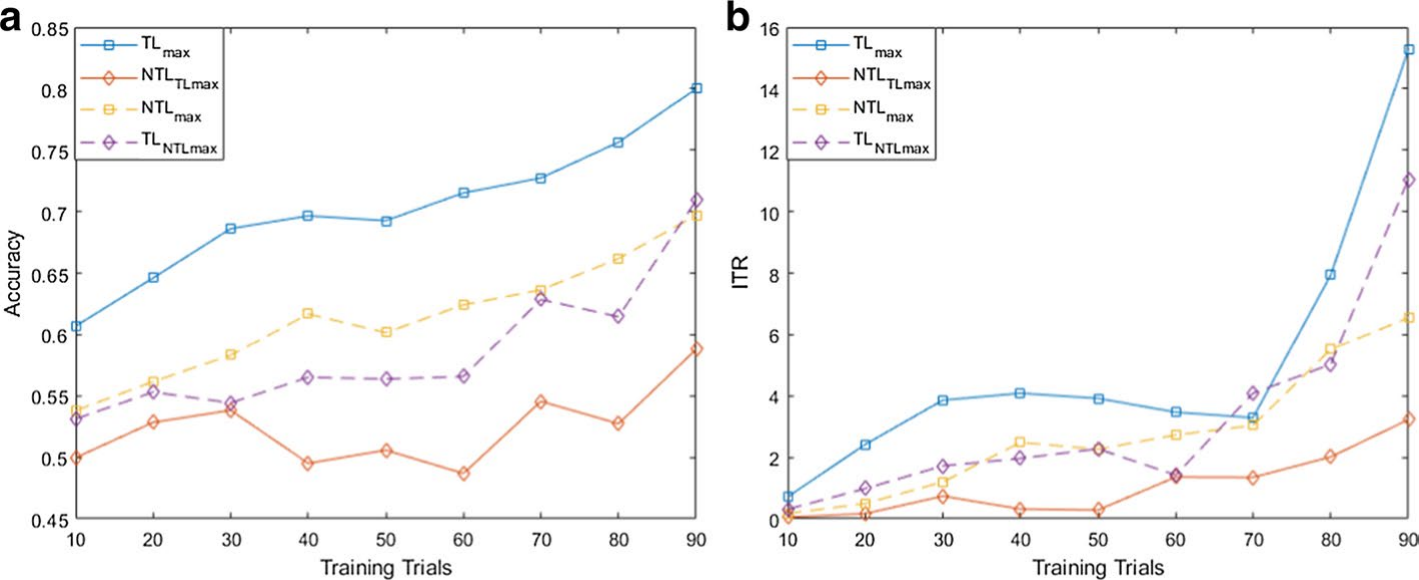
Average accuracy (**a**) and average ITR (**b**) as a function of the number of training trials MR versus WG problem

**Fig. 6:**
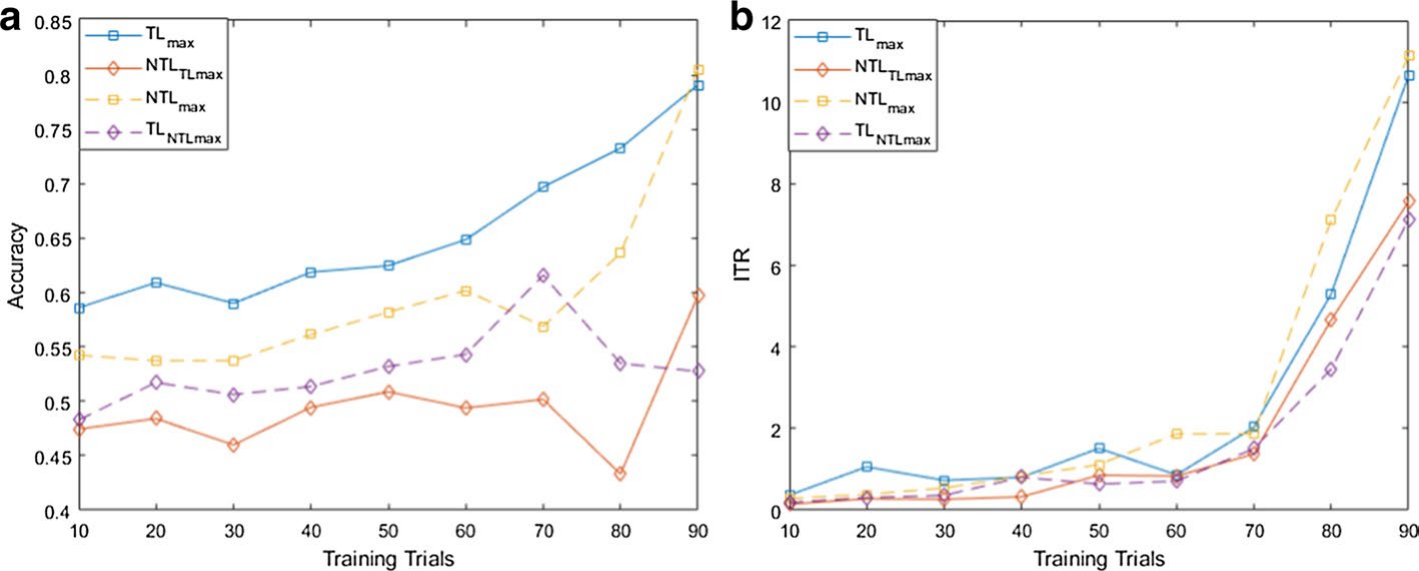
Average accuracy (**a**) and average ITR (**b**) as a function of the number of training trials for WG versus baseline problem

For both MI and flickering MR/WG paradigms, to evaluate the effectiveness of the proposed TL approach, for each binary selection problem, we reported the average accuracies and ITRs across participants obtained using different training set sizes. Moreover, we compare these accuracies/ITRs with those obtained without transfer learning (NTL). Figures 1, 2, 3, 4, 5 and 6 reflect the impact of the amount of data available to train a prediction model on the accuracy/ITR that can be obtained with and without transfer learning. In particular, the *x*-axis shows the number of training trials, ranging from 10 to 90 trials, used to train a prediction model, while the *y* axis shows the average accuracy/ITR across participants corresponding to those training trials.

For both TL and NTL cases, at each training set size, a classifier is trained, and its performance is evaluated for each participant at trial lengths of 1, 2…10 s. The maximum accuracy/ITR at each training set size is reported regardless of the corresponding trial length. The average accuracy/ITR is computed across all participants at different training set sizes. Therefore, in terms of calibration requirements, comparing the best possible performances obtained for TL and NTL cases are not entirely fair since these performances are not evaluated at the same calibration length. In particular, calibration length is not only a function of the number of training trials, but also a function of trial length which varies depending on when maximum accuracy/ITR could be achieved. Therefore, as seen in Figs. 1, 2, 3, 4, 5 and 6, to ensure fair comparison, in addition to reporting the best possible TL and NTL performances, we evaluated the performance of NTL at the same trial lengths that yield the maximum possible TL performance. In addition, we evaluated the performance of TL and the same trial lengths that yield the maximum NTL performance.

### MI paradigm

As seen in Figs. 1, 2 and 3, TL performance evaluated at the trial lengths that yield the maximum NTL performance is similar to maximum NTL performance while the performance of NTL at the same trial lengths that yield the maximum possible TL performance is significantly worse than the maximum TL performance. Disregarding differences in trial length, average accuracies obtained using TL are significantly higher than those obtained without transfer learning (NTL) as shown in Figs. 1, 2 and 3. Moreover, in terms of ITRs, it can be also noted that TL provides the highest ITRs compared to NTL case.

In addition, we observed that, when TL is employed, using only 10 training trials, average accuracies of 80.58%, 75.29%, and 69.16% can be achieved for right MI versus baseline, left MI versus baseline, and right MI versus left MI, while for NTL case, the average accuracies that can be obtained using 10 training trials are 56.63%, 58.14%, and 60.21%, respectively. In terms of ITRs, at 10 training trials, it can be noted that right MI versus baseline, left MI versus baseline, and right MI versus left MI achieved average ITRs of 2.34, 2.13, and 2.98 bits/min, respectively, compared to 1.51, 0.54, and 1.74 bits/min obtained for NTL case.

Using 90% of the available data for training which corresponds to 90 training trials, TL achieved accuracies of 98.89%, 98.00%, and 94.67% and ITRs of 16.5, 20.51, and 11.3 bits/min for right MI versus baseline, left MI versus baseline, and right MI versus left MI, respectively, compared to of accuracies of 80.00%, 78.33%, and 76.67% and ITRs of 7.83, 7.04, and 6.27 bits/min achieved without TL.

Using (15), we found that the calibration requirements for MI paradigm can be reduced by 80.00%, 60.43%, and 81.99% for right MI versus baseline, left MI versus baseline, and right MI versus left MI, respectively.

### Flickering MR/WG paradigm

Figures 4, 5 and 6 show that TL performance evaluated at the trial lengths yielding the maximum NTL performance is comparable to maximum NTL performance while the performance of NTL at the same trial lengths yielding the maximum possible TL performance is significantly worse than maximum TL performance. Disregarding trial length, for the 3 binary selection problems, average accuracy and ITR trends obtained using TL are significantly higher than those obtained without transfer learning (NTL) especially at smaller training set sizes as shown in Figs. 4, 5 and 6. However, for WG versus baseline problem, we observed that ITRs obtained using TL outperform those obtained without TL for training set sizes < 50 trials.

We observed also that when the training set size drops to 10 trials, transfer learning provides an improvement in the accuracy by approximately 11%, 5%, and 7% for MR versus baseline, WG versus baseline, and MR versus WG. In terms of ITRs, at 10 training trials, 1, 0.37, and 0.71 bits/min were obtained for MR versus baseline, WG versus baseline, and MR versus WG using TL, while without TL, 0.28, 0.29, and 0.17 bits/min were achieved for the same classification problems.

Using 90 training trials, TL achieved 82.83%, 79.09%, and 80.00% average accuracies and 8.13, 10.66, and 15.28 bits/min average ITRs MR for versus baseline, WG versus baseline, and MR versus WG, respectively, while NTL obtained 75.76%, 80.52%, and 69.97% average accuracies and 6.83, 11.13, 6.55 bits/min average ITRs for the same classification problems.

Using (15), we found that the calibration requirements for flickering MR/WG paradigm can be reduced by 17.31% and 12.96% for MR versus baseline and MR versus WG, respectively, while for WG versus baseline, TL approach only boosted the performance accuracy without reducing the calibration requirement.

## Discussion

For MI paradigm, it can be concluded that, using 10 training trials, TL can improve the average performance accuracy by 9–24% for the 3 binary selection problems compared to NTL case, while using 100% of the available training data (90 trials), performance of NTL case can be enhanced by 18–20% for the 3 classification problems. Moreover, ITRs obtained using TL at 10 training trials are 1.8–2.90 times the ITRs obtained without TL, while at 90 training trials, ITRs of TL case are 1.5–3.94 times the ITRs obtained without TL.

As for MR/WG paradigm, at 10 training trials, improvements ranging from 5 to 11% in average accuracy as well as ITRs that are 1.28–4.18 times ITRs of NTL case can be achieved for MR versus baseline and MR versus WG. At 90 training trials, performance can be enhanced by 7–10% average accuracy with 1.19–2.33 times ITRs of NTL case. However, there is no improvement in performance for WG versus baseline problem when using 100% of the available training data.

Comparing the average accuracies and ITRs obtained using both paradigms as well as their average accuracy and ITR improvements compared to NTL case especially at 10 training trials, it can be concluded that the proposed transfer learning algorithm is more efficient when used with MI paradigm. Therefore, TL can be used to reduce the calibration requirements of the system while maintaining sufficient performance that is comparable to NTL performance with a higher number of training trials. For instance, given only 10 training trials from the current BCI user who uses MI paradigm, accuracies ranging from 70 to 80% can be achieved for the 3 classification problems when using the proposed transfer learning approach. This corresponds to a maximum of 100 s calibration length.

Considering the trade-off between the calibration length and the corresponding BCI performance, it is the BCI designer’s decision to choose the optimal number of trials to be recorded from each BCI user to calibrate the system. Given that the proposed transfer learning approach has significantly reduced the calibration requirements of the MI-based hybrid BCI by at least 60.43%, we believe that our proposed approach gives more flexibility to the BCI designers to control and reduce the calibration requirements of the system which is an important criterion especially when the BCI is intended to be used by patients with disabilities.

## Conclusion

In this paper, aiming at decreasing the calibration requirements of our hybrid EEG–fTCD BCI as well as improving its performance, we propose a transfer learning approach that identifies the top similar datasets to the current BCI user and combines the trials from these datasets as well as few training trials from the current user to train a classifier that can predict the test trials of that user with high accuracy. To achieve such aim, EEG and fTCD feature vectors of each trial were projected into two scalar SVM scores. EEG and fTCD class conditional distributions were learnt separately using the scores of each class. Bhattacharyya distance was used to identify similarities between class conditional distributions obtained using training trials of the current BCI user and those obtained using trials of previous BCI users. Experimental results showed that the performance obtained using the proposed transfer learning approach outperforms the performance obtained without transfer learning for both MI and flickering MR/WG paradigm. However, comparing performance improvement achieved for both paradigms, it can be noted that the proposed transfer learning algorithm is more efficient when used with MI paradigm. In particular, average accuracies and ITRs of 80.58%, 75.29%, and 69.16% and 2.34, 2.13, and 2.98 bits/min can be achieved for right MI versus baseline, left MI versus baseline, and right MI versus left MI using 10% of the available data which corresponds to a calibration length of 100 s. Moreover, it was found that the calibration requirements of MI paradigm can be reduced by at least 60.43% when using the proposed transfer learning approach.

## Materials and methods

### Data acquisition

A g.tec EEG system was employed for EEG data acquisition using 16 EEG electrodes positioned at locations Fp1, Fp2, F3, F4, Fz, Fc1, Fc2, Cz, C1, C2, Cp3, Cp4, P1, P2, P5, and P6. Reference electrode was placed over left mastoid. The collected data were sampled with 256  samples/s sampling rate. Moreover, data were filtered using the g.tec amplifier’s bandpass filter (corner frequencies: 2 and 62 Hz) and the amplifier’s notch filter with 58 and 62 Hz corner frequencies.

fTCD data collection was performed using a SONARA TCD system with two 2 MHz transducers placed on the right and left sides of the transtemporal window which is located above the zygomatic arch [17]. Given that middle cerebral arteries (MCAs) are responsible of approximately 80% of brain blood perfusion [18], the fTCD depth was set to the depth of the mid-point of the MCAs which is 50 mm [19].

### Presentation paradigms

**Fig. 7:**
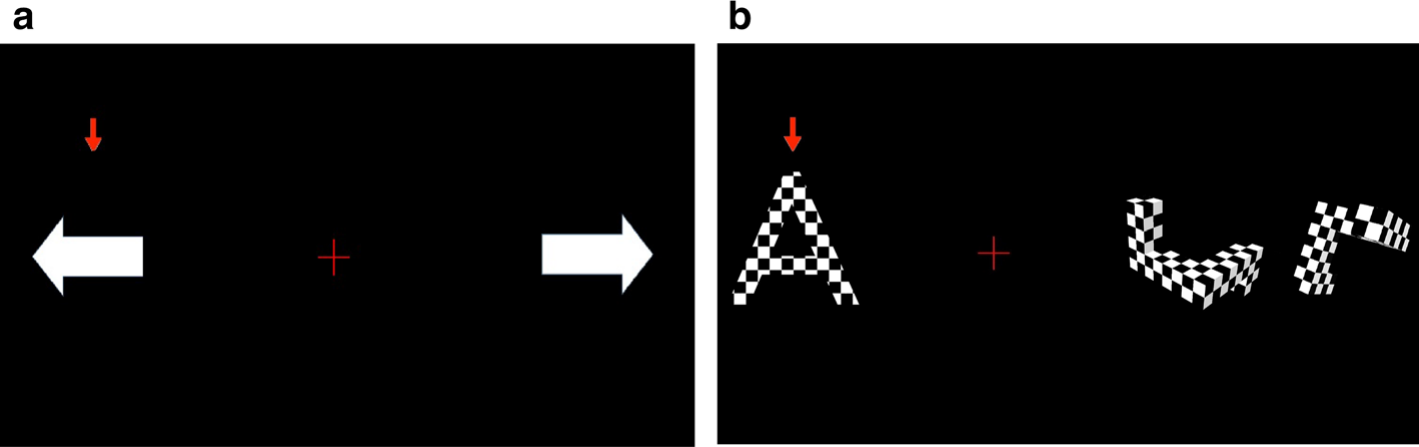
Stimulus presentation for our motor imagery EEG–fTCD BCI (**a**) and the proposed flickering MR/WG hybrid BCI (**b**)

We designed two different presentation paradigms to be used with the proposed hybrid BCI. The first paradigm employed motor imagery (MI) tasks while the other paradigm used flickering mental rotation (MR) and word generation (WG) tasks as shown in Fig. 7. For both paradigms, while acquiring EEG and fTCD simultaneously, two tasks and a fixation cross that represents the baseline were presented on the screen. Total of 150 trials were presented to each user and during each trial, a vertical arrow randomly selected one of the three visual icons representing the two tasks and the baseline. The vertical arrow pointed to the selected icon for 10 s and the user was asked to perform the task identified by that arrow until the arrow points to another visual icon.

During the MI-based presentation scheme, a basic MI task was presented to the users as shown in Fig. 7a. In particular, a horizontal white arrow that points to the right represented right arm MI while a horizontal white arrow that points to the left represented left arm MI. The baseline was represented by the fixation cross shown in the middle [20].

During MR/WG presentation paradigm, since MR and WG tasks are known to be differentiated using fTCD only, to make them differentiable in terms of EEG, the visual icons of MR and WG tasks were textured with a flickering checkerboard pattern as seen in Fig. 7b and they flickered at 7 Hz and 17 Hz, respectively, to induce different SSVEPs in EEG [21]. During WG task, the user was asked to silently generate words starting with the letter shown on the screen while during MR task, the user was given two 3D shapes and was asked to mentally rotate one of these shapes and decide if they were identical or mirrored.

### Participants

The local Institutional Review Board (IRB) of University of Pittsburgh approved all the study procedures (IRB number: PRO16080475). All the subjects were consented before starting the experiment. A total of 21 healthy individuals participated in this study. In particular, to assess flickering MR/WG paradigm, data were collected from 11 individuals (3 females and 8 males) with ages ranging from 25 to 32 years while, to test MI paradigm, data were collected from 10 subjects (4 males and 6 females) with ages ranging from 23 to 32 years. None of the subjects participated in the study had a history of heart murmurs, concussions, migraines, strokes, or any brain-related injuries. Each subject attended one session that included 150 trials and each trial lasted for 10 s.

### Feature extraction

In this section, we describe our feature extraction approaches applied to EEG and fTCD signals collected using both MI and flickering MR/WG paradigm.

#### EEG

##### MI paradigm

We employed common spatial pattern (CSP) to analyze EEG MI data [6]. CSP is known to be an efficient feature extraction technique for MI-based EEG BCIs as it can extract EEG spatial patterns that characterize different MI tasks [22]. CSP aims at finding a linear transformation that changes the variance of the observations representing two different classes such that the two classes are more separable [23]. More specifically, CSP learns the optimal spatial filters that result in maximizing the variance of one class in a certain direction, and in the mean time, minimize the variance of the second class in the same direction [24]. These filters can be found by solving the following optimization problem:

1 $$\text{max} _{W} {\text{tr}}\;W^{T} \varSigma_{c} W$$

$${\text{s}}.{\text{t}}. \, W^{T} \left( {\varSigma_{\left( + \right)} + \varSigma_{\left( - \right)} } \right)W = 1,$$

where $\varSigma_{c}$ is the average trial covariance for class $c \epsilon \left\{ { + , - } \right\}$ and $W$ the transformation matrix.

Assume each trial data are represented as a matrix $R^{N \times T}$ where $N$ is the number of EEG electrodes and $T$ represents the number of the samples for each electrode. Sample covariance of each trial $m$ can be calculated as follows:

2 $$S_{m} = \frac{{{\text{RR}}^{T} }}{{{\text{tr}}\left( {{\text{RR}}^{T} } \right)}}.$$

Using (2), the average trial covariance can be calculated as given below

3 $$\sum_{c} = \frac{1}{M}\mathop \sum \limits_{m = 1}^{M} S_{m} ,$$

where $M$ is the number of trials belonging to class $c$.

(1) is solved through simultaneously diagonalizing the covariance matrices $\varSigma_{c}$ which can be represented as follows:

4 $$\begin{aligned} W^{{}} \varSigma_{\left( + \right)} W = \varLambda_{\left( + \right)} \hfill \\ W^{T} \varSigma_{\left( - \right)} W = \varLambda_{\left( - \right)} , \hfill \\ {\text{s}}.{\text{t}}. \, \varLambda_{\left( + \right)} + \varLambda_{\left( - \right)} = I \hfill \\ \end{aligned}$$

where $$\varLambda_{c}$$ is a diagonal matrix with eigenvalues $$\lambda_{j}^{c} ,$$$$j = 1,2,3, \ldots N$$ on its diagonal.

Solution of (4) is similar to the solution of the generalized eigenvalue problem below:

5 $$\varSigma_{\left( + \right)} w_{j} = \lambda \varSigma_{\left( - \right)} w_{j}$$

where $w_{j}$ is the $j{\text{th}}$ generalized eigenvector and $\lambda = \frac{{\lambda_{j}^{\left( + \right)} }}{{\lambda_{j}^{\left( - \right)} }}$. (4) is satisfied when the transformation matrix is equivalent to $W = \left[ {w_{1} ,w_{2} , \ldots w_{N} } \right]$ and $\lambda_{j}^{c}$ is given by

6 $$\lambda_{j}^{c} = w_{j}^{T} \varSigma_{c} w_{j}$$

where $\lambda_{j}^{c}$ are the elements on diagonal of $\varLambda_{c}$. $\lambda_{j}^{\left( + \right)} + \lambda_{j}^{\left( - \right)} = 1$, since $\varLambda_{\left( + \right)} + \varLambda_{\left( - \right)} = I$.

It can be noted that a higher value of $\lambda_{j}^{\left( + \right)}$ will result in a higher variance in the data representing class $c = +$ when filtered using $w_{j}$. Given that a high value of $\lambda_{j}^{\left( + \right)}$ results in a low $\lambda_{j}^{\left( - \right)}$ value, when filtering the data of class $c = -$ using $w_{j}$, a low variance will be obtained. In this study, we solved 3 binary MI selection problems by considering different numbers of eigenvectors. More specifically, MI EEG data were spatially filtered using 1, 2, …., and 8 eigenvectors from both ends of the transformation matrix $\left( W \right)$. For each trial, log variance of each filtered signal was computed and considered as a feature.

##### MR/WG paradigm

As explained before in our previous study, we used template matching to extract features from EEG data [5]. More specifically, for each class, since each trial is represented by 16 EEG segments collected from 16 electrodes, we extract 16 templates corresponding to the 16 EEG electrodes by averaging EEG training trials over each electrode. To extract features representing each trial, cross-correlations between the segments of that trial and the corresponding 16 templates representing each class were calculated. Maximum cross-correlation score across each of 16 cross-correlations was considered as a feature resulting in a total of 16 features. Given that the problems of interest are binary classification problems, the feature vector representing each trial contained a total of 32 features.

#### fTCD

5-level wavelet decomposition [25] was used to analyze the two fTCD data segments of each trial with Daubechies 4 mother wavelet. To decrease the fTCD feature vector dimensions, instead of considering each wavelet coefficient as a feature, we calculated statistical features for each of the 6 wavelet bands resulting from the wavelet analysis. These features included mean, variance, skewness, and kurtosis [26, 27]. Therefore, each trial was represented by 24 features for each fTCD data segment and a total of 48 features.

### Feature selection and projection

Wilcoxon rank-sum test [28] with a *p* value of 0.05 was employed for the selection of the significant features from both EEG and fTCD feature vectors of MR/WG paradigm while it was used to select only fTCD significant features of MI paradigm. As for MI EEG, the feature vector representing a certain trial was composed of $2f$ features obtained through transforming the data of the trial using $f =$ 1, 2, …., and 8 eigenvectors from both ends of the transformation matrix $\left( W \right)$.

EEG and fTCD feature vectors of each trial were then projected separately into 2 SVM scalar scores (EEG and fTCD evidences). To evaluate the performance of both MI and MR/WG paradigms, these evidences were combined under the Bayesian fusion approach explained in “Bayesian fusion and decision making” section. Performance of the MI hybrid system was evaluated using $2f$ (2, 4,…, and 16) EEG CSP features. The highest performance measures obtained with and without transfer learning were reported and compared in the results section while for MR/WG paradigm, performance measures with and without transfer learning were calculated and compared only at *p* value of 0.05.

### Bayesian fusion and decision making

We developed a Bayesian fusion approach of EEG and fTCD evidences to infer user intent at a given trial considering three different assumptions [5, 6]. Under assumption ($A1$), EEG and fTCD evidences are assumed to be jointly distributed while under assumption ($A2$), EEG and fTCD evidences are assumed to be independent. Under assumption ($A3$), evidences of EEG and fTCD are assumed to be independent, but they contribute unequally toward taking a right decision. For each binary selection problem, tenfold cross validation was used to define training and testing trials. Our previous work showed that the best performance was achieved under assumption $A3$ for MI paradigm; therefore, for MI, we utilized the assumption *A*3 in this paper [6]. On the other hand, for flickering WG/MR paradigm, *A*2 and $A3$ both had high performance without any statistically significant differences [5]. However, $A3$ is more computationally complex compared to $A2$; therefore, for WG/MR paradigm, we performed probabilistic fusion under assumption $A2$ [5].

Given that $N$ trials are introduced to each participant, these trials are represented by a set of EEG and fTCD evidences $Y = \left\{ {y_{1} , \ldots y_{N} } \right\}$ where $y_{k} = \left\{ {e_{k} , f_{k} } \right\}$, $e_{k}$ and $f_{k}$ are EEG and fTCD evidences of a test trial $k$. User intent $x_{k}$ for the test trial $k$ can be inferred through joint state estimation using EEG and fTCD evidences which can be represented as follows:

7 $$\widehat{{x_{k} }} = \arg \mathop {\text{max} }\limits_{{x_{k} }} p\left( {x_{k} |Y = y_{k} } \right)$$

where $p(x_{k} |Y)$ is the state posterior distribution conditioned on the observations $Y$. Using Bayes rule, (7) can be rewritten as

8 $$\widehat{{x_{k} }} = \arg \mathop {\text{max} }\limits_{{x_{k} }} p(Y = y_{k} |x_{k} ) p\left( {x_{k} } \right)$$

where $p(Y|x_{k} )$ is the state conditional distribution of the measurements $Y$ and $p\left( {x_{k} } \right)$ is the prior distribution of user intent $x_{k}$. Since the trials are randomized, $p\left( {x_{k} } \right)$ is assumed to be uniform. Therefore, (8) can be reduced to

9 $$\widehat{{x_{k} }} = \arg \mathop {\text{max} }\limits_{{x_{k} }} p(Y = y_{k} |x_{k} ).$$

$p(Y|x_{k} )$ of each class can be estimated using the EEG and fTCD evidences computed for the training trials. To infer user intent at a test trial $k$, Eq. (9) is solved at $Y = y_{k} .$ Here, the distributions $p(Y|x_{k} = 1)$ and $p(Y|x_{k} = 2)$ are evaluated under two assumptions as explained below.

#### Assumption 2: independent distributions

Here, the evidences of EEG and fTCD, conditioned on $x_{k}$, are assumed to be independent. Therefore, (9) can be rewritten as

10 $$\widehat{{x_{k} }} = \arg ,\mathop {\text{max} }\limits_{{x_{k} }} p(e = e_{k} |x_{k} )p(f = f_{k} |x_{k} )$$

where $p(e|x_{k} )$ and $p(f|x_{k} )$ are the distributions of EEG and fTCD evidences conditioned on the state $x_{k}$ respectively. To find $p(e|x_{k} )$ and $p(f|x_{k} )$, kernel density estimation (KDE) with Gaussian kernel was employed using evidences of EEG and fTCD of the training trials. Kernel bandwidth was computed using Silverman’s rule of thumb [29]. $e_{k}$ and $f_{k}$ are plugged in (10) to infer the user intent of a test trial $k$ where the user intent $x_{k}$ that maximizes the likelihood is selected.

#### Assumption 3: weighted independent distributions

Here, we assume that evidences of EEG and fTCD are independent, but they contribute unequally toward taking a right decision. Therefore, we propose weighting $p(e|x_{k} )$ and $p(f|x_{k} )$ conditional distributions with weights of $\alpha$ and $1 - \alpha$, respectively. (9) can be rewritten as

11 $$\widehat{{x_{k} }} = \arg ,\mathop {\text{max} }\limits_{{x_{k} }} p(e = e_{k} |x_{k} )^{\alpha } p(f = f_{k} |x_{k} )^{1 - \alpha }$$

where $\alpha$ is a weighting factor ranging from 0 to 1. $p(e|x_{k} )$ and $p(f|x_{k} )$ are computed as mentioned in “Assumption 2: independent distributions” section. Finding the optimal *α* value is performed through applying a grid search over $\alpha$ values ranging between 0 and 1 with a step of 0.01.

### Transfer learning algorithm

With the aim of decreasing calibration requirements and improving the performance of the hybrid system, we propose a transfer learning approach that identifies the top similar datasets collected from previous BCI users to a training dataset collected from a current BCI user and uses these datasets to augment the training data of the current BCI user. The proposed transfer learning approach is intended to be used for both MI and flickering MR/WG paradigms. Therefore, the performance of the proposed approach was tested using the 6 binary selection problems of both paradigms.

#### Similarity measure

To apply transfer learning to a certain binary selection problem, for each dataset from previous BCI users, EEG and fTCD feature vectors of trials corresponding to that problem were projected into scalar SVM scores. Therefore, each trial was represented by a scalar EEG SVM score and a scalar fTCD SVM score. Using KDE, 2 EEG class conditional distributions and 2 fTCD class conditional distributions were learnt from these scores. KDE was performed using Gaussian kernel. EEG and fTCD class conditional distributions of the current BCI user were also estimated using his/her training trials.

To measure the similarity between the class conditional distributions of the current BCI user and those of the previous users, Bhattacharyya distance [30], given by (12), was used since it is a symmetric measure that can be applied to general distributions especially if these distributions are diverging from normal distributions and it provides bounds on Bayesian misclassification probability, which overall fits very well to our approach of making Bayesian decisions on binary classification problems using the estimated density functions.

12 $$d = - \ln \mathop \sum \limits_{i = 1}^{N} P_{i} Q_{i} ,$$

where $P$ and $Q$ are 2 probability distributions and $N$ is the number of points composing each distribution.

Bhattacharyya distance between EEG class conditional distribution of class $i \left( {i = 1, 2} \right)$ and the corresponding EEG class conditional distribution of the current BCI user was calculated. Bhattacharyya distance was also calculated between the fTCD class conditional distributions of each previous BCI user and the current BCI user. Sum of these 4 distances (2 EEG distances and 2 fTCD distances) represented the total distance between the current BCI user and a certain previous BCI user.

#### Proposed transfer learning algorithm

**Fig. 8:**
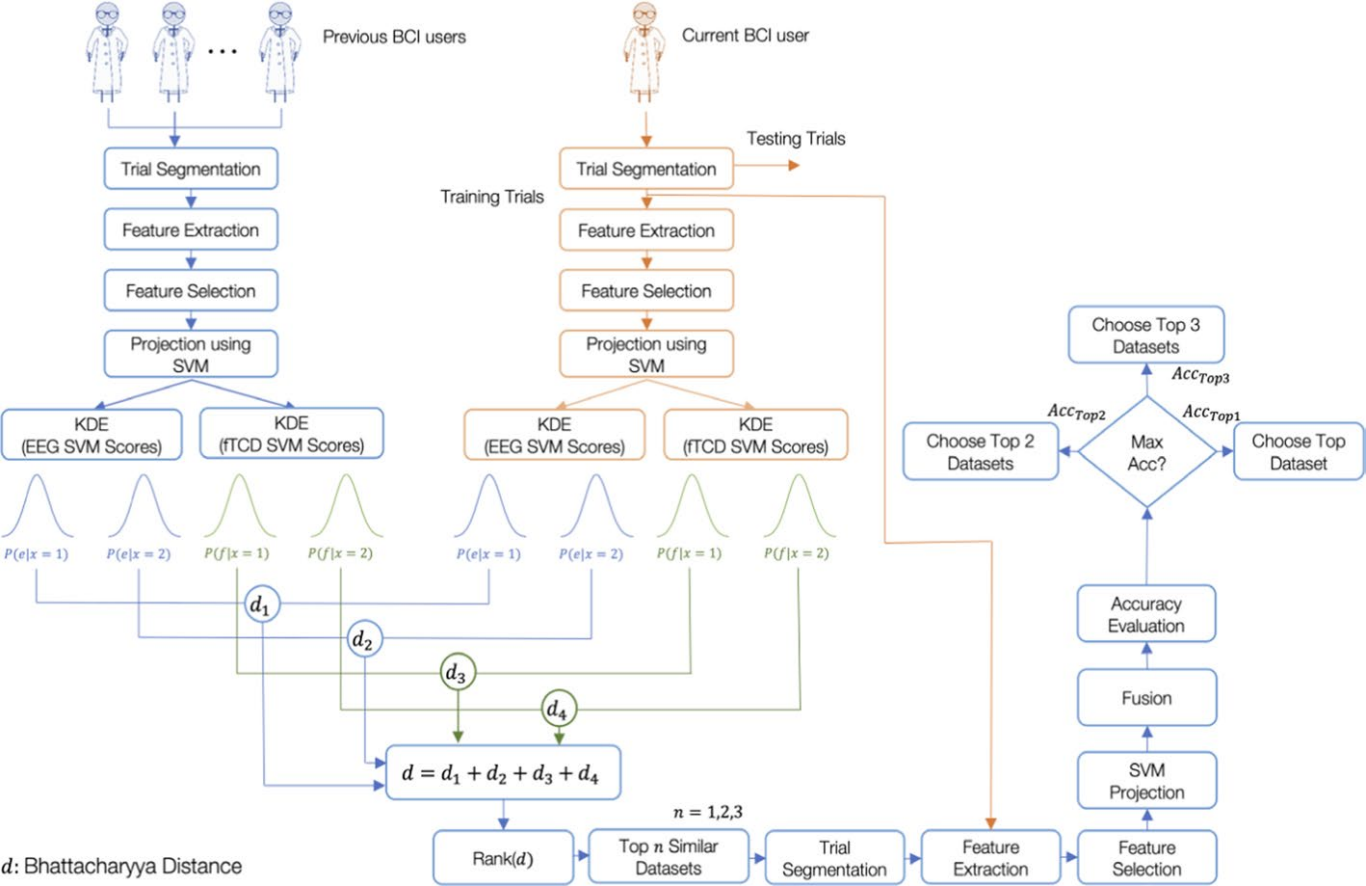
Identification of the top similar datasets using the proposed transfer learning approach

**Fig. 9:**
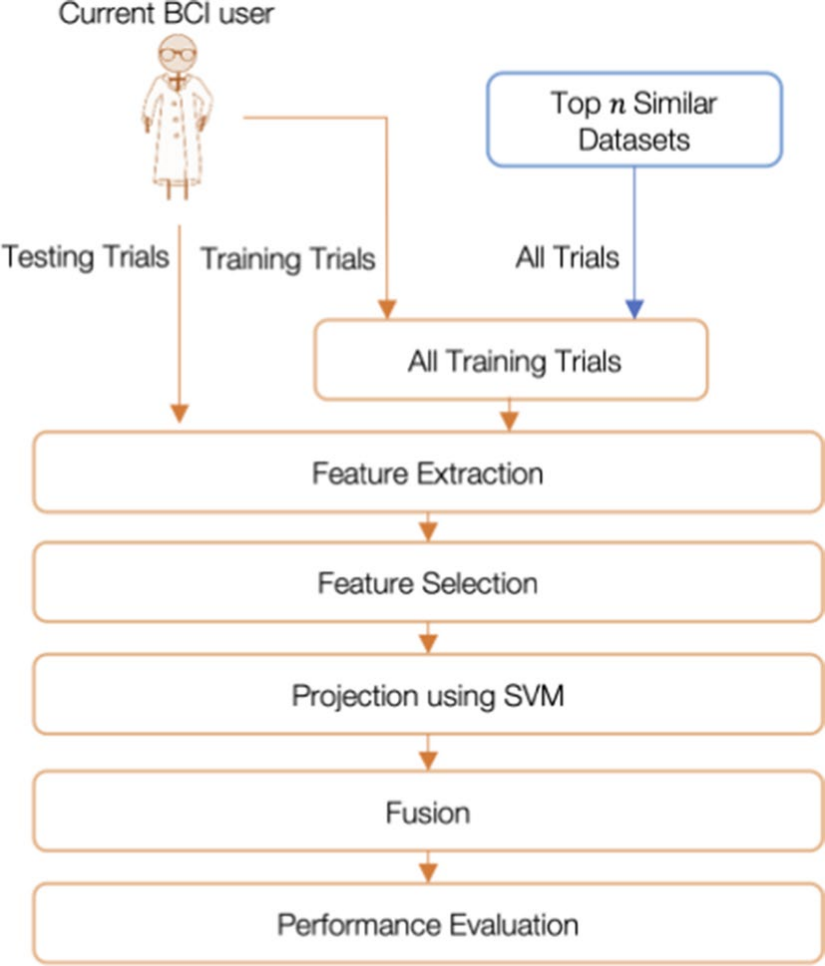
Testing phase of the proposed transfer learning approach

The proposed transfer learning approach is described in detail in Figs. 8 and 9. Given a group of previous BCI users where each user is represented by one dataset, the objective is to find the most similar datasets to the training dataset of the current BCI user and to combine the trials from these datasets with small number of training trials from the current user to train a classifier that can predict the labels of the test trials of that user with high accuracy. In particular, for each binary selection problem, the dataset of the current user was divided into training and testing sets. Initially, given that each binary selection problem is represented by 100 trials, we used the first 10 trials from the current BCI user for training the prediction model and the remaining 90 trials for testing. As seen in Fig. 8, features are extracted from training trials of the current user as well as the trials corresponding to the binary problem of interest from each of the previous BCI users. Extracted EEG and fTCD features vary depending on the paradigm used for data collection. In particular, CSP and wavelet decomposition were used to extract features from the data of the MI paradigm while template matching and wavelet decomposition were used to extract features from the data of the flickering MR/WG paradigm as explained in “Feature extraction” section. After applying the feature selection step detailed in “Feature selection and projection” section, EEG and fTCD feature vectors of each trial were projected into 2 scalar SVM scores.

For each class within the binary selection problem of interest, we learnt class conditional distributions of the EEG and fTCD scores obtained from SVM projection as seen in Fig. 8. Distance between class conditional distributions of the current BCI user and those of each of the previous BCI users was computed as explained in “Similarity measure” section. To identify the top similar datasets, these distances were sorted ascendingly. At this point, it was required to decide on how many similar datasets should be considered to train the classifier besides the training trials from the current BCI user. Here, we considered a maximum of 3 datasets to be combined with the training trials of the current BCI user. Through crossvalidation, the number of top similar datasets that maximize the performance accuracy when combined with the training trials of the current user was chosen to be used later to predict test trials of the current BCI user as shown in Fig. 9. Here, for each participant, we used up to 3 datasets to be used for transfer learning. However, the maximum number of datasets could be increased or decreased depending on the needs of the designers. Moreover, the presented framework could be used to identify person-specific maximum number of datasets. For future versions of this algorithm, instead of using a maximum of 3 datasets to be combined with the training trials of the current BCI, such number can be optimized for each subject separately by means of model order selection techniques [31].

To study the impact of the training set size (from the current BCI user) on the performance of the proposed transfer learning approach, we applied the proposed approach on training sets of size ranging from 10 to 90 trials which corresponds to test sets of size ranging from 90 to 10 trials.

### Performance evaluation

For both MI and flickering MR/WG paradigms, to assess the significance of the transfer learning (TL) compared to the no transfer learning case (NTL), for each participant, accuracy and information transfer rate (ITR) [32] were calculated and compared at different number of training trials from the current BCI user. In particular, at every number of training trials, accuracy and ITR were calculated at time points 1, 2….,10 s. For each number of training trials, maximum accuracy and ITR across the 10-s trial length were reported for TL and NTL cases. ITR can be calculated as follows:

13 $$B = \log_{2} \left( N \right) + P\log_{2} \left( P \right) + \left( {1 - P} \right)\log_{2} \left( {\frac{1 - P}{N - 1}} \right)$$

where *N* represents the number of BCI selections, *P* represents the classification accuracy, and *B* is the information transfer rate per trial.

To compute the reduction in calibration requirements for each binary problem when using TL compared to NTL case, at each training set size, we formed a vector containing performance accuracies obtained for all participant at that training set size. We statistically compared the accuracy vectors of TL at training set sizes of 10, 20…,90 with accuracy vector obtained for NTL case at maximum training set size (90 trials). Initially, at 10 training trials, we performed one-sided Wilcoxon signed rank test between the accuracy vector of TL with 10 training trials and NTL accuracy vector at 90 training trials. Such statistical comparison is repeated with TL applied at bigger training set sizes until there is no statistically significant difference between the performance of TL and the performance of NTL at 90 trials. The number of trials $N$ at which that statistical insignificance occurs is used in (14) to compute percentage of reduction.

14 $${\text{Reduction}}\% = \frac{1}{P}\mathop \sum \limits_{i = 1}^{P} \frac{{{\text{Calibration length}}_{\text{NTL}} \left( i \right) - {\text{Calibration}}\;{\text{length}}_{\text{TL}} \left( i \right)}}{{{\text{Calibration length}}_{\text{NTL}} \left( i \right)}} \times 100\% .$$

Equation (14) is equivalent to

15 $${\text{Reduction\% }} = \frac{1}{P}\mathop \sum \limits_{i = 1}^{P} \frac{{N \times {\text{Trial length}}_{N} \left( i \right)_{\text{NTL}} - m \times {\text{Trial length}}_{m} \left( i \right)_{\text{TL}} }}{{N \times {\text{Trial length}}_{N} \left( i \right)_{\text{NTL}} }} \times 100{\text{\% }}$$

where $N$ is the maximum number of training trials ($N$ = 90) from the current BCI user and $m$ is the minimum number of trials at which TL performance is at least equivalent to NTL performance where $m$ ranges from 10 to 90 trials.

To guarantee that TL will improve or at least achieve the same average performance accuracy obtained for the NTL case, we checked if the TL average performance accuracy at $m$ training trials was similar to or outperforms the average performance accuracy of NTL case at 90 training trials. If this condition is not satisfied, we consider statistical comparisons at training set sizes $> m$ until this condition is satisfied.

## Data Availability

The datasets used and/or analysed during the current study are available on reasonable request.

## Abbreviations

BCI: Brain–computer interface
CSP: Common spatial pattern
EEG: Electroencephalography
fNIRS: Functional near-infrared spectroscopy
fTCD: Functional transcranial Doppler
ITR: Information transfer rate
LSPA: Limited speech and physical abilities
MR: Mental rotation
NTL: No transfer learning
SNR: Signal to noise ratio
SVM: Support vector machines
TL: Transfer learning
WG: Word generation
